# Microwave-assisted CVD-like synthesis of dispersed monolayer/few-layer N-doped graphene encapsulated metal nanocrystals for efficient electrocatalytic oxygen evolution†
†Electronic supplementary information (ESI) available. See DOI: 10.1039/c8sc02444h


**DOI:** 10.1039/c8sc02444h

**Published:** 2018-07-20

**Authors:** Fanxing Bu, Wenshu Chen, Jiajun Gu, Phillips O. Agboola, Najeeb Fuad Al-Khalli, Imran Shakir, Yuxi Xu

**Affiliations:** a State Key Laboratory of Molecular Engineering of Polymers , Department of Macromolecular Science , Fudan University , Shanghai 200433 , China . Email: xuyuxi@fudan.edu.cn; b State Key Laboratory of Metal Matrix Composites , Shanghai Jiao Tong University , Shanghai , 200240 , China; c Mechanical Engineering Department , College of Applied Engineering , King Saud University (Al Muzahimiyah Branch) , Riyadh , Saudi Arabia; d Department of Electrical Engineering , King Saud University , Riyadh 11421 , Kingdom of Saudi Arabia; e Sustainable Energy Technologies Center , College of Engineering Center , King Saud University , Riyadh 11421 , Kingdom of Saudi Arabia . Email: imranskku@gmail.com

## Abstract

Herein a novel and general microwave-assisted chemical vapor deposition (CVD)-like synthetic strategy was developed to realize the ultrafast synthesis of a series of well-dispersed monolayer/few-layer N-doped graphene shell encapsulated metal nanocrystals (M@NC) by using a metal–organic framework (MOF) on graphene as precursors for the first time. Unlike traditional programmed heat treatment, this microwave-assisted method decomposed the MOF into separated metal and carbon- and nitrogen-containing gases rather than aggregated metal and carbon composites during the initial thermal transformation stages. This change ensured the effective control of the subsequent formation process of carbon on the surface of metal and led to the formation of well-dispersed M@NC with monolayer/few-layer NC. Moreover, the graphene substrate promoted the full exposure of all active monolayer/few-layer NC, and thus the obtained FeNi@NC/graphene displays the best electrocatalytic properties for the oxygen evolution reaction of all of the previously reported M@NC based catalysts, including the lowest overpotential (261 mV) at 10 mA cm^–2^ in alkaline electrolyte (1 M KOH), the smallest Tafel slope (40 mV dec^–1^) and excellent durability for at least 120 h.

## Abstract 2

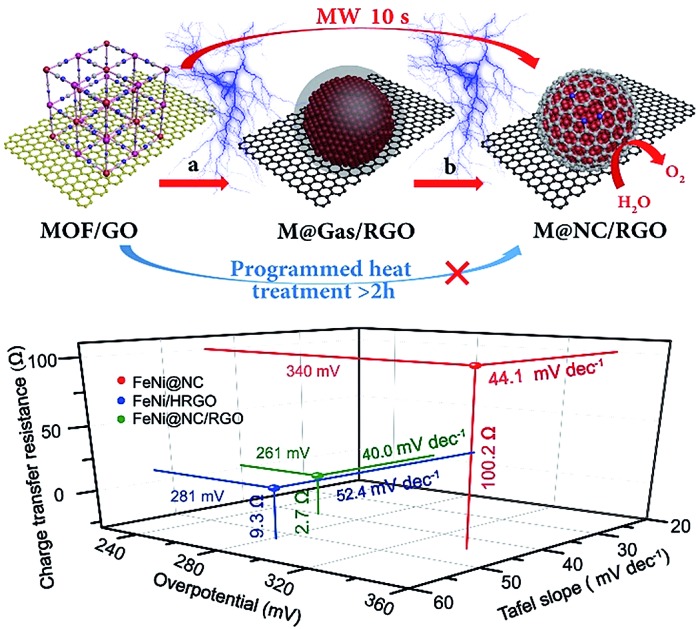
A novel CVD-like synthetic strategy was developed to realize ultrafast synthesis of a series of monolayer/few-layer N-doped graphene encapsulated metal nanocrystals with excellent electrocatalytic oxygen evolution performance.

## Introduction

Core–shell metal or alloy@graphene nanocrystals (M@C)^1^–^5^ are unique members of the graphitic carbon-based composite family that separate functional materials from the external environment, which is quite different from those graphene and carbon nanotubes-based composites that usually expose other functional materials on their surface.^6^ The encapsulation of graphene on metal not only enhances the stability of metal nanocrystals by preventing them from gas and solution corrosion, but also constructs a unique functional M/C interface with a controllable electronic structure.^7^–^14^ Therefore, M@C have been developed as some of the most promising electrocatalysts with decent activity, excellent stability, especially in harsh reaction conditions such as strong acidic or alkaline reaction media, and high overpotential in electrocatalysis. Due to highly adjustable metal compositions, the electronic structure of graphene has been facilely mediated to endow M@C with excellent electrocatalytic properties for a range of reactions including the hydrogen evolution reaction (HER), the oxygen evolution reaction (OER), and the oxygen reduction reaction (ORR).^7^–^16^ Currently, one of the most important scientific tasks involves the pushing of their electrocatalytic performances to those of the state-of-the-art noble metal-based electrocatalysts. Recently, it was demonstrated by density functional theory (DFT) calculations that two strategies could be employed to enhance the electrocatalytic properties of M@C. One is heteroatom doping such as N-doping in the graphene shell, which could increase the density of state (DOS) near the Fermi level. The other one is decreasing the layer number of the graphene shell, which could promote electron transfer from the metal to the graphene shell.^7^–^16^ Thus it is highly attractive to fabricate N-doped monolayer/few-layer graphene encapsulated metal (M@NC) nanocrystals and explore their highly enhanced electrocatalytic performances.

Currently, traditional programmed thermal decompositions of metal-, carbon- and nitrogen-containing precursors, especially metal–organic frameworks (MOFs), are the main routes to synthesize M@NC.^7^,^8^,^11^–^16^ It is widely recognized that metal ions are reduced into metal nanocrystals, and organic ligands are decomposed into carbon and/or maybe other carbon- and nitrogen-containing gases firstly, and then carbon on the surface of the metal is transformed into N-doped graphene.^17^–^19^ However, due to the uncontrollable aggregation of metal catalytic sites and diffusion of the carbon source during the decomposition process of the MOF under traditional programmed heat treatment, the obtained products are usually discrete metal nanocrystals encapsulated within carbon nanotubes and/or pomegranate-shaped M@NC aggregations with relatively thick graphene shells. These undesirable structures unavoidably lead to the formation of NC sites with low activity, as well as a decrease in the exposure of active NC sites, and this thus leads to undesirable catalytic activity. Moreover, long high-temperature reaction times and huge amounts of inert gas consumption greatly increase the costs and limit large-scale production. Therefore, it is still a great challenge to realize the facile synthesis of M@NC with monolayer/few-layer NC, and new synthetic strategies based on the controllable reaction of metal and carbon sources are urgently needed.

**Fig. 1:**
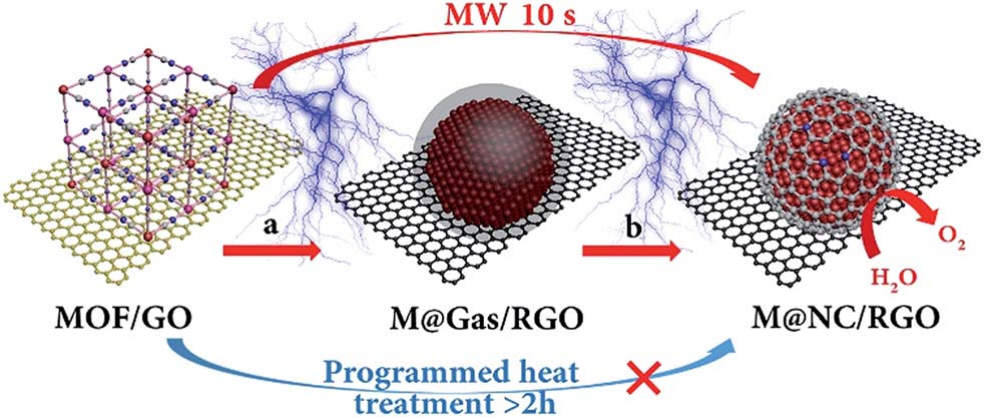
Schematic illustration of the ultrafast microwave-assisted CVD-like synthesis of M@NC/RGO. (a) The decomposition of a MOF into metal and carbon- and nitrogen-containing gases, (b) CVD-like deposition of the NC on the surface of the metal nanocrystals.

In this work, a versatile ultrafast microwave-assisted chemical vapor deposition (CVD)-like synthetic route was developed to convert MOF on graphene oxide (GO) into well-dispersed M@NC nanocrystals with few-layer NC on reduced GO (M@NC/RGO), within 10 seconds, in the presence of carbon cloth (CC). Different from traditional programmed heat treatment, CC and RGO act as microwave susceptors^20^,^21^ to create prodigious amounts of heat rapidly under microwave irradiation, which directly decompose the MOF deposited on the GO into separated metal nanocrystals and carbon- and nitrogen-containing gases rather than aggregated metal nanocrystals and carbon composites (Fig. 1a). This ensures a CVD-like synthesis of M@NC nanocrystals with few-layer NC through the deposition of these gases on the surface of the metal (Fig. 1b). The composition of the metal nanocrystals could be facilely adjusted by changing the composition of the MOF, and a series of M@NC/RGO including FeNi@NC/RGO, CoNi@NC/RGO, Co@NC/RGO and FeCoNi@NC/RGO have been successfully fabricated. Integrating the higher activity of the few-layer NC with sufficient exposure of all active sites, the obtained FeNi@NC/RGO could behave as excellent OER electrocatalysts. They not only display the lowest overpotential (261 mV) at 10 mA cm^–2^ in alkaline electrolyte (1 M KOH) and the smallest Tafel slopes (40 mV dec^–1^) ever reported for all M@NC based catalysts, but also could achieve 50 and 100 mA cm^–2^ currents with the very low overpotentials of 287 and 299 mV. Moreover, the catalysts could be operated at different current densities for at least 30 h without any obvious degradation.

## Results and discussion

**Fig. 2:**
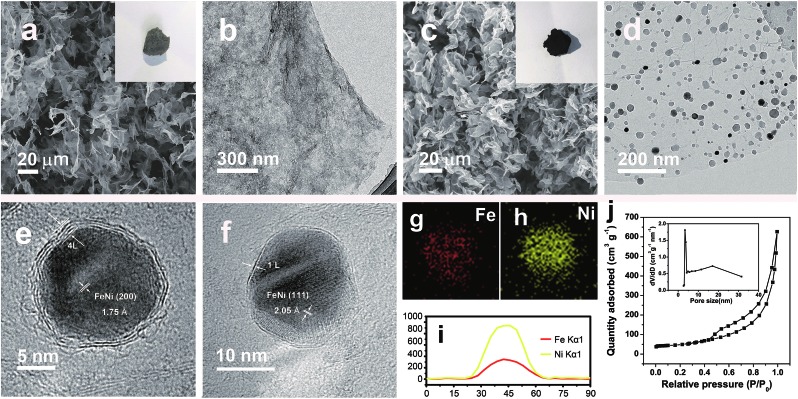
(a) SEM and (b) TEM images of Ni–Fe PBA/GO. (c) SEM, (d) TEM and (e and f) HRTEM images of FeNi@NC/RGO, (inset in a and c: photographs of Ni–Fe PBA/GO and FeNi@NC/RGO sponges). (g and h) EDS mapping pictures and (i) line profiles of Fe and Ni recorded on a single FeNi@NC nanoparticle. (j) N_2_ sorption isotherm of FeNi@NC/RGO, (inset: BJH pore size distribution curve).

Prussian blue analogues (PBAs) are one kind of classical MOF that are self-assembled from metal ions and cyanide ligands that can be facilely prepared by co-precipitation of metal cations (M^*x*+^) and cyanometalate anions ([N(CN)_*y*_]^*z*–^, *x* = 2 or 3, *y* = 4 or 6, *z* = 2, 3, or 4, M and N = Fe, Co, Ni, Mn, or Cr) in water solution.^22^–^24^ The highly tunable metal components, and the nitrogen-containing ligands, as well as the low cost, have made PBAs the most widely used precursors for the fabrication of M@NC. Here FeNi@NC derived from nickel ferrocyanide (Ni–Fe PBA) was chosen as a model to demonstrate our concept firstly. Specifically, Ni–Fe PBA was first grown *in situ* on GO to form the PBA/GO composite (Fig. S1†) by the excessive metal ion induced self-assembly strategy that developed by us recently.^24^ After freeze-drying, the obtained porous Ni–Fe PBA/GO sponge, observed by scanning electron microscopy (SEM) Fig. 2a, was treated with microwave irradiation under Ar gas in the presence of CC. When light electric arc appeared, the color of the sponge changed to black immediately and the FeNi@NC/RGO sponge (Fig. 2c) was obtained after 10 s. It should be noted that CC is necessary for this rapid conversion reaction. In the absence of CC, this reaction could not be initiated, even after microwave irradiation for 20 min. Similar to previous reports,^20^,^21^ CC serves as a microwave susceptor to catalyse the conversion of GO to RGO in the early reaction stages. Then RGO acts as a microwave susceptor to ensure the uniform thermal conversion reaction of all MOFs on RGO. As shown in Fig. 2b, one layer of Ni–Fe PBA nanoparticles, with sizes smaller than 20 nm, cover the whole surface of the GO in the Ni–Fe PBA/GO composite. After microwave radiation, discreet FeNi@NC nanocrystals, with an average size of about 20 nm, that are well dispersed on RGO are successfully obtained (Fig. 2d). High-resolution transmission electron microscopy (HRTEM) images (Fig. 2e and f) clearly demonstrated that FeNi alloy nanocrystals were well encapsulated within layered graphitized NC with a typical interplanar distance of about 0.34 nm. It should be noted that these were different from those M@NC obtained from traditional programmed heat treatment methods,^11^–^13^ and the layer numbers of most NC in this work were smaller than 5 layers, and 1 layered NC were frequently observed (Fig. 2g). This is advantageous for electron transfer from the metal to the surface of the NC and promotes their catalytic properties.^9^,^16^ Meanwhile, the representative lattice fringe spacings of 1.75 Å and 2.05 Å agree well with the crystal lattice of the (200) and (111) planes of the cubic FeNi alloy phase.^9^,^13^ Consistent and uniform distribution of Fe and Ni in Energy-dispersive X-ray spectroscopy (EDS) elemental mapping images (Fig. 2g and h) and line profiles (Fig. 2i) further confirm that the obtained FeNi was a phase pure alloy rather than a mixture of Fe and Ni. In addition, the N_2_ sorption analysis of the FeNi@NC/RGO composite (Fig. 2j) demonstrates that the FeNi@NC/RGO had a Brunauer–Emmett–Teller (BET) surface area of 167.6 m^2^ g^–1^, and the Barrett–Joyner–Halenda (BJH) pore size distribution curve displays that the FeNi@NC/RGO possesses a mesoporous structure. The high surface area and the mesoporous structure not only expose a large amount of surface active sites but also facilitate guest transport, which also benefits the electrochemical properties.^25^,^26^

**Fig. 3:**
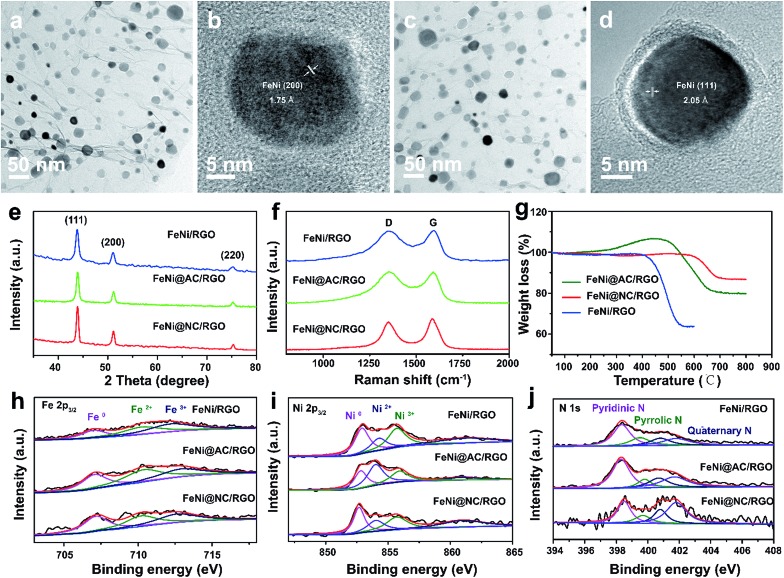
(a and b) TEM images of FeNi/RGO obtained by microwave irradiation for 2 s; (c and d) TEM images of FeNi@AC/RGO obtained by microwave irradiation for 5 s; (e) XRD, (f) Raman and (g) TGA spectra, and XPS spectra of (h) Fe 2p_3/2_, (i) Ni 2p_3/2_ and (j) N 1s of FeNi/RGO, FeNi@AC/RGO and FeNi@NC/RGO.

The simple and ultrafast fabrication process and the desirable few-layer NC structure indicate that the FeNi@NC/RGO materials are formed by a new mechanism that is different from that of traditional programmed heat treatment. Therefore, time-dependent experiments during the formation process of FeNi@NC were conducted. As shown in Fig. 3a–d, pure FeNi dispersed on RGO (FeNi/RGO) and amorphous carbon coated FeNi deposited on RGO (FeNi@AC/RGO) formed as the dominant products after microwave treatment for 2 s and 5 s. That is to say, under microwave irradiation, Ni–Fe PBA was probably directly decomposed into separated metal and carbon- and nitrogen-containing gases rather than the aggregated metal and carbon composites present in traditional programmed heat treatment. Then, those gases were redeposited on the surface of the metal and transformed into layered graphitized NC by a CVD-like synthesis, catalyzed by FeNi. This is feasible since a high temperature up to 1500 °C could be created by microwave irradiation,^20^ which could be verified by the rapid evaporation of NaCl in this reaction system. In this case, the aggregation of FeNi nanocrystals was largely depressed by RGO, which promoted the exposure of more of the metal surface and led to the formation of few-layer NC. X-ray diffraction analysis (XRD), Raman, thermogravimetric analysis (TGA) and X-ray photoelectron spectroscopy (XPS) analysis were carried out to further reveal the transformation process. As shown in Fig. 3e, those products formed after 2 s of microwave irradiation were already cubic FeNi alloys^9^,^13^ and the composition had no obvious change during the following reaction time, indicating that this reaction is indeed an ultrafast process. The Raman spectra in Fig. 3f demonstrate that the *I*_G_/*I*_D_ ratios for FeNi/RGO, FeNi@AC/RGO and FeNi@NC/RGO are 1.01, 0.99 and 1.11 respectively. Theoretically, the *I*_G_/*I*_D_ ratio should increase gradually with the reaction time since high temperatures promote the removal of defects in graphene, and the lowest value for FeNi@AC/RGO is likely to be caused by the existence of redeposited AC. In addition, the TGA results in Fig. 3g display that the loading mass of the FeNi alloy in FeNi/RGO, FeNi@NC/RGO and FeNi@NC/RGO is gradually increased from 44.6% to 56.3% and then 61.2% (see characterizations in the ESI†). Moreover, they demonstrate different decomposition behaviors. As we know, during the oxidative decomposition process of these products, oxidation of FeNi will increase the mass, while that of RGO would decrease the mass. In addition, high quality graphene with a low amount of defects should have a high decomposition temperature. Thus it could be reasonably deduced that the oxidations of FeNi and RGO occurred simultaneously for FeNi/RGO at relatively low temperature, and the oxidation of FeNi occurs earlier than the decomposition of RGO in FeNi@AC/RGO. A slight mass increase takes place before the decomposition of RGO, while the encapsulation of FeNi by NC largely delays the oxidation of FeNi and the obvious mass loss is observed at relatively high temperature for FeNi@NC/RGO. Lastly, XPS of FeNi/RGO and FeNi@NC/RGO was used as further characterization to examine the electron state of the metal and the nitrogen. It was clearly observed that the 2p_2/3_ peaks of Fe and Ni (Fig. 3h and i) could be deconvoluted into three peaks assigned to M^0^ (around 707.1 eV for Fe and 852.4 eV for Ni), M^2+^ (around 710.3 eV for Fe^2+^ and 853.9 eV for Ni^2+^) and M^3+^ (around 712.9 eV for Fe^3+^ and 855.8 eV for Ni^3+^).^12^,^13^ Obviously, the encapsulation of the metal with graphitized NC prevents the adsorption of oxygen species on the metal and increase the content of M^0^, which was confirmed by the semi-quantitative analysis based on the peak area ratios (Table S1†). In addition, the N 1s peaks (Fig. 3j) could all be divided into four peaks attributed to pyridinic N (398.4 eV), pyrrolic N (399.5 eV and 400.8 eV) and quaternary N (401.7 eV).^12^,^13^ As displayed in Fig. 3j and Table S1,† the content of quaternary N increased from FeNi/RGO to FeNi@NC/RGO, indicating that more perfect RGO formed over a longer reaction time, which is in consistent with the above Raman results. It should be noted that the well-dispersed M@NC nanocrystals with few-layer graphitized NC shells could not be obtained by traditional programmed heat treatment whether using Ni–Fe PBA/GO or Ni–Fe PBA as precursors (Fig. S2†). As shown in Fig. S2a–c,† even though the Ni–Fe PBA on GO was transformed into FeNi alloy after traditional programmed heat treatment at 700 °C under Ar for 2 h, no obvious graphitized NC was observed on the surface of the FeNi alloy and lot of holes appeared on the RGO (denoted as FeNi/HRO). It is possible that some of the Ni–Fe PBA nanoparticles reacted with GO to form metal oxides at low temperature and then the metal oxides were reduced by RGO to form FeNi alloy dispersed on holey RGO, which could be confirmed by the formation of a mixture of metal oxide and cubic metal at 600 °C (Fig. S2a†). In the absence of GO, aggregates of FeNi alloy with sizes ranging from dozens of nanometers to hundreds of nanometers encapsulated with thick and obvious carbon shells (denoted as FeNi@NC) were the dominant products (Fig. S2d–f†). The N_2_ desorption analysis in Fig. S3† demonstrates that the FeNi@NC had a BET surface area of 44.1 m^2^ g^–1^. This value is four times lower than that of FeNi@NC/RGO, demonstrating that the obtained FeNi@NC/RGO might have more available active NC sites. The above results fully demonstrate that well-dispersed FeNi@NC nanocrystals with few-layer NC were fabricated by a novel ultrafast microwave-assisted CVD-like synthetic route, which could not be obtained by traditional programmed heat treatment.

**Fig. 4:**
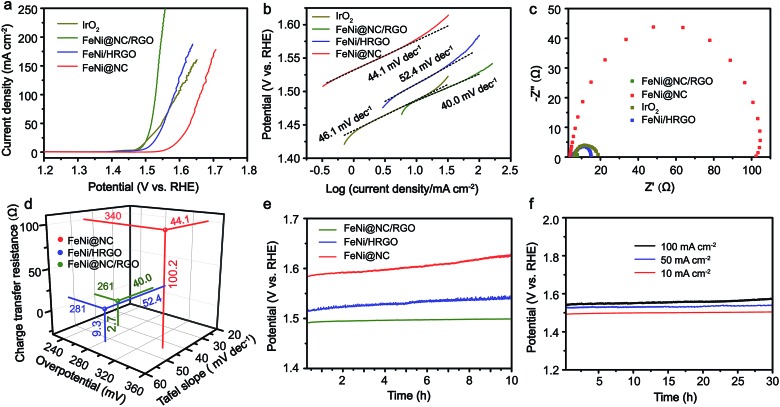
(a) *iR*-Corrected polarization curves, (b) Tafel curves, (c) EIS characterizations, (d) overpotentials, Tafel slopes and charge transfer resistances, and (e) OER CP curves at 10 mA cm^–2^ of FeNi@NC/RGO, FeNi/HRGO, FeNi@NC and IrO_2_. (f) OER CP curves of FeNi@NC/RGO at 10, 50 and 100 mA cm^–2^.

Inspired by the advantageous structure of FeNi@NC/RGO, the electrocatalytic properties of the composite in OER were evaluated in a 1 M KOH solution by using a typical three-electrode electrochemical cell with Hg/HgO and graphite rod electrodes used as the reference electrode and the counter electrode, respectively. The catalytic performances of FeNi@HRGO and FeNi@NC obtained by programmed heat treatment, and IrO_2_ were also examined as references. As shown in Fig. 4a–d, the FeNi@NC/RGO shows a very low overpotential of 261 mV to reach 10 mA cm^–2^ and a very small Tafel slope of 40.0 mV dec^–1^, indicating that FeNi@NC/RGO possesses highly active reaction sites and also ultrafast OER kinetics.^27^–^30^ Moreover, ultrahigh current densities of 50, 100, 150, 200, 250 mA cm^–2^ could be obtained at the overpotentials of 287, 299, 307, 317 and 326 mV, which is highly encouraging for its practical application. In contrast, the overpotentials of FeNi@HRGO and FeNi@NC to achieve 10 mA cm^–2^ were 281 and 340 mV, and the Tafel slopes were 52.4 and 44.1 mV dec^–1^ (Fig. 4a–d), which are much higher than that of FeNi@NC/RGO. Even though IrO_2_ could achieve 10 mA cm^–2^ at an overpotential of 257 mV, its mass loading (10.4 mg cm^–2^) was 20 times higher than that of FeNi@NC/RGO. Moreover, the Tafel slope of IrO_2_ (46.1 mV dec^–1^) was lower than that of FeNi@NC/RGO. Lower charge transfer resistance often means higher OER activity of FeNi@NC/RGO and a faster reaction rate during the catalytic process.^26^,^29^ The better performance of FeNi@NC/RGO could be attributed to the smallest charge transfer resistance of about 2.7 Ω, which is smaller than those of FeNi/HRGO (9.3 Ω), FeNi@NC (100.2 Ω) and IrO_2_ (12.0 Ω).

**Fig. 5:**
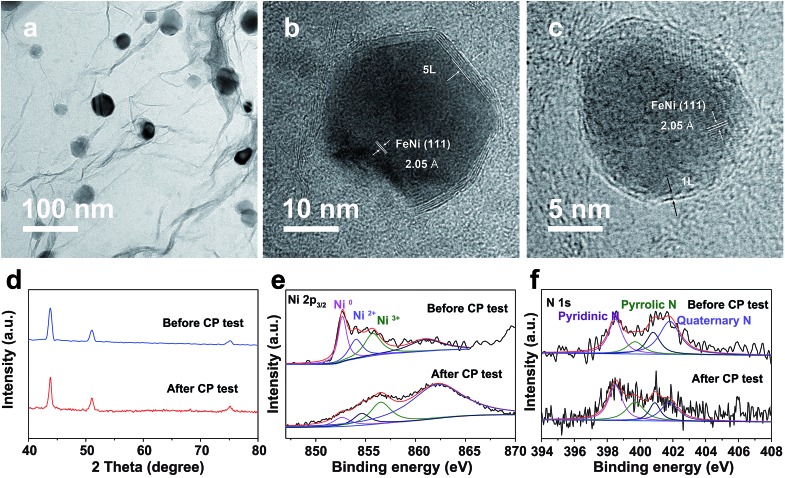
(a) TEM and (b and c) HRTEM images, (d) XRD images and XPS spectra of Ni 2p_3/2_ (e) and N 1s (f) of FeNi@NC/RGO after OER CP testing at 10 mA cm^–2^ for 10 h.

Notably, even in 0.1 M KOH, the FeNi@NC/RGO could also achieve 10 and 50 mA cm^–2^ at very low overpotentials of about 292 and 378 mV (Fig. S4a†). The corresponding Tafel slope is quite low, only 65.5 mV dec^–1^ (Fig. S4b†). In addition, FeNi@NC/RGO also demonstrates the best long-term cycling stability as exhibited in Fig. 4e. After 10 h of OER chronopotentiometry (CP) test at a current of 10 mA cm^–2^, the overpotential of FeNi@NC/RGO only increased by 7.4 mV while those of FeNi@HRGO and FeNi@NC increased by 28.5 mV and 41.1 mV, respectively. Even with prolonged CP tests at 10 mA cm^–2^ for 30 and 120 h, only slight overpotential increases of about 10.9 mV and 20.1 mV were observed for FeNi@NC/RGO (Fig. S5†). Moreover, FeNi@NC/RGO could be operated at 50 and 100 mA cm^–2^ for at least 30 h of CP testing with only slight overpotential increases of about 20.2 and 31.5 mV, respectively (Fig. 4f). The above results demonstrate the excellent OER stability of FeNi@NC/RGO, which could be further verified by post-characterizations of the catalyst. As shown in Fig. 5a–c, good structural stability of the FeNi@NC/RGO composite was confirmed by the observation of intact graphitized NC shells and clear lattice fringes of the FeNi alloy after CP tests. Then, the compositional stability was proven by similar XRD images before and after CP tests (Fig. 5d). In addition, XPS results showed that the intensity of oxidized metal increased after CP tests (Fig. 5e), which might be caused by the increased adsorption of oxygen species on the surface of FeNi@NC/RGO induced by the high OER potential and/or a slightly oxidized surface of some of the FeNi alloy that not completely encapsulated.^9^,^13^ The four peaks assigned to pyridinic N, pyrrolic N and quaternary N were all be observed in the N 1s spectra after CP tests and only little changes in their peak intensities took place (Fig. 5f), further verifying the stability of FeNi@NC/RGO during OER tests. To the best of our knowledge, this is the best M@NC-based OER catalyst in alkaline electrolyte, and its catalytic properties are also close to the best results of other materials shown in Table S2.† According to the above analysis, the remarkable catalytic performance of the FeNi@NC/RGO composite could be ascribed to the following few points. Firstly, the microwave-assisted CVD-like synthetic method ensure the successful synthesis of FeNi@NC with few-layer carbon shells, which should promote the charge transfer from the FeNi alloy to the NC and endow them with intrinsically high catalytic activity.^9^,^16^ Secondly, the RGO prevents the aggregation of FeNi nanocrystals during the formation process. This not only contributes to the formation of more active NC sites, but also ensures the sufficient use of all of those active sites. At the same time, the mesoporous structure could facilitate mass transport and might accelerate the OER reaction.^25^,^26^ Lastly, the high electro-conductivity promoted by intimately contacted RGO, metal and graphitic carbon ensure fast electron transport.

**Fig. 6:**
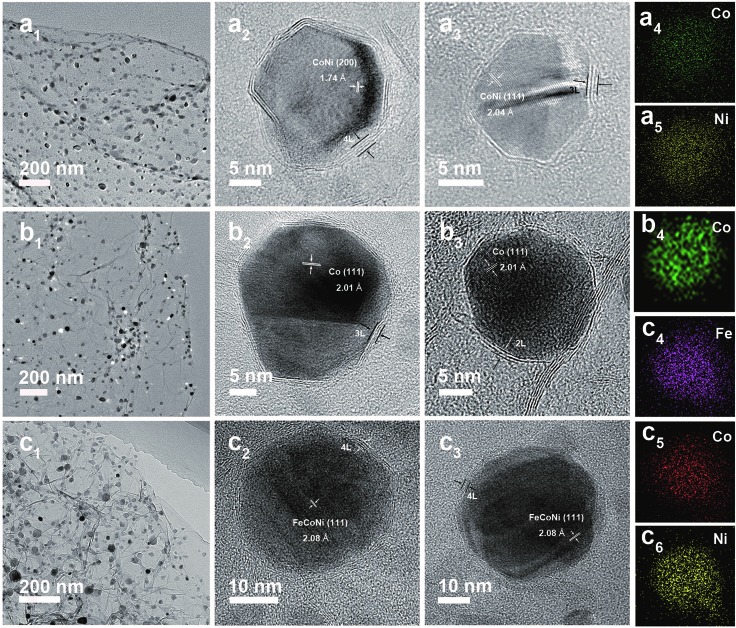
TEM and HRTEM images of CoNi@NC/RGO (a_1_–a_3_), Co@NC/RGO (b_1_–b_3_) and FeCoNi@NC/RGO (c_1_–c_3_). EDS mapping images of single CoNi@NC (a_4_ and a_5_), Co@NC (b_4_) and FeCoNi@NC (c_4_–c_6_) nanocrystals.

**Fig. 7:**
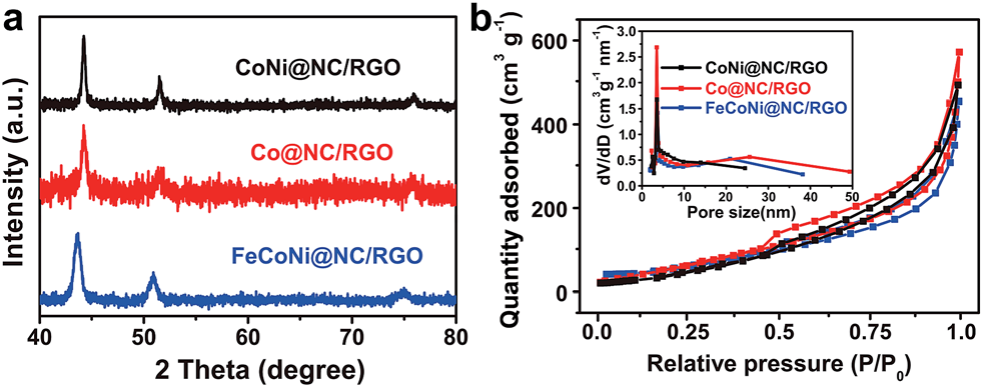
(a) XRD images and (b) N_2_ sorption isotherms of M@NC/RGO, (inset: BJH pore size distribution curves).

The low-cost ultrafast CVD-like synthetic strategy, well-dispersed core@shell structure with few-layer NC, as well as the extraordinary OER performance of FeNi@NC/RGO encouraged us to explore the capability of this method for fabricating other M@NC materials, since M@NC materials with different metal components could perform as excellent electrocatalysts for different applications.^7^–^16^ As shown in Fig. 6 and 7a, not only other binary alloy CoNi but also pure metal Co and the ternary alloy, FeCoNi, could be encapsulated with few-layer NC on RGO by using face-centered cubic (FCC) structured nickel hexacyanocobaltate (Ni–Co PBA), cobalt hexacyanocobaltate (Co–Co PBA) and cobalt/nickel hexacyanocobaltate (CoNi–Fe PBA) nanocrystals with sizes smaller than 20 nm deposited on GO as precursors (Fig. S6–S9†).^9^,^13^ The average sizes of CoNi@NC, Co@NC and FeCoNi@NC nanocrystals were 20 nm, 20 nm and 19 nm, respectively (Fig. 6a_1_–c_1_). The core@shell structures of those composites were clearly confirmed by HRTEM analysis of the M@NC nanocrystals as shown in Fig. 6a_2_–c_2_, a_3_–c_3_ and S7–S9.† The clear lattice fringes could be assigned to (111) and (200) planes of the cubic phase metals and alloys. Similar to FeNi@NC/RGO, the NC shells were very thin, usually <5 layers. In addition, the N_2_ desorption analysis (Fig. 7b) demonstrated that they had similar N_2_ desorption isotherms to FeNi@NC/RGO. The obvious hysteresis loop at *P*/*P*_0_ > 0.4 indicates that they all have mesoporous structures, which could be demonstrated by their BJH pore size distributions between 2 nm and 50 nm. The BET surface areas of CoNi@NC/RGO, Co@NC/RGO, and FeCoNi@NC/RGO were 120.8, 184.2 and 177.6 m^2^ g^–1^, respectively. The above results indicated that M@NC with other metal components that have similar structures to FeNi@NC could also be synthesized by the microwave-assisted CVD-like method, fully confirming the versatility of this unique method. This not only presents a solid foundation for the construction of other M@NC composites, but also largely promotes their potential in commercial processes.

## Conclusions

In conclusion, a new and general ultrafast microwave-assisted thermal transformation route (<10 s) has been developed for the construction of well-dispersed core@shell M@NC nanocrystals with monolayer/few-layer NC by using MOF/GO composites as precursors. It was revealed that CC and RGO act as microwave susceptors to create huge amounts of heat, and then transform the MOF into core@shell M@NC nanocrystals by a CVD-like formation mechanism. Unlike traditional programmed heat treatment, this method could effectively control the deposition of NC on the surface of the metal and then promoted the maximized formation of an active monolayer/few-layer NC. As a result, the obtained FeNi@NC/RGO displays the lowest overpotential (261 mV) at 10 mA cm^–2^ in alkaline electrolyte (1 M KOH), the smallest Tafel slope (40.0 mV dec^–1^) and excellent durability for at least 120 h, compared to all previous M@NC based OER catalysts. Encouragingly, the method is so versatile that M@NC structures with pure metal, binary alloy and ternary alloy could all be achieved. This simple and controlled synthesis of M@NC with tailorable structures and properties will promote the understanding of thermal conversion science of MOFs and the development of various MOF-derived functional materials.

## Conflicts of interest

There are no conflicts to declare.

## Supplementary Material

Supplementary information

## References

[cit1] Ruoff R. S., Lorents D. C., Chan B., Malhotra R., Subramoney S. (1993). Science.

[cit2] Dravid V. P., Host J. J., Teng M. H., Hwang B. E. J., John-son D. L., Mason T. O., Weertman J. R. (1995). Nature.

[cit3] Jiao J., Seraphin S. (1998). J. Appl. Phys..

[cit4] Liang Y.-C., Hwang K. C., Lo S.-C. (2008). Small.

[cit5] Hsin Y.-L., Lin C.-F., Liang Y.-C., Hwang K. C., Horng J.-C., Ho J.-A. A., Lin C.-C., Hwu J. R. (2008). Adv. Funct. Mater..

[cit6] Huang X., Qi X., Boey F., Zhang H. (2012). Chem. Soc. Rev..

[cit7] Deng D., Yu L., Chen X., Wang G., Jin L., Pan X., Deng J., Sun G., Bao X. (2013). Angew. Chem., Int. Ed..

[cit8] Zheng X., Deng J., Wang N., Deng D., Zhang W.-H., Bao X., Li C. (2014). Angew. Chem., Int. Ed..

[cit9] Cui X., Ren P., Deng D., Deng J., Bao X. (2016). Energy Environ. Sci..

[cit10] Tavakkoli M., Kallio T., Reynaud O., Nasibulin A. G., Jo-hans C., Sainio J., Jiang H., Kauppinen E. I., Laasonen K. (2015). Angew. Chem., Int. Ed..

[cit11] Yang Y., Lun Z., Xia G., Zheng F., He M., Chen Q. (2015). Energy Environ. Sci..

[cit12] Su J., Yang Y., Xia G., Chen J., Jiang P., Chen Q. (2017). Nat. Commun..

[cit13] Yang Y., Lin Z., Gao S., Su J., Lun Z., Xia G., Chen J., Zhang R., Chen Q. (2017). ACS Catal..

[cit14] Wang J., Gao D., Wang G., Miao S., Wu H., Li J., Bao X. (2015). Nano Energy.

[cit15] Jia G., Zhang W., Fan G., Li Z., Fu D., Hao W., Yuan C., Zou Z. (2017). Angew. Chem., Int. Ed..

[cit16] Deng J., Yu L., Deng D., Chen X., Yang F., Bao X. (2013). J. Mater. Chem. A.

[cit17] Kaneti Y. V., Tang J., Salunkhe R. R., Jiang X., Yu A., Wu K. C. W., Yamauchi Y. (2017). Adv. Mater..

[cit18] Aparicio C., Machala L., Marusak Z. (2012). J. Therm. Anal. Calorim..

[cit19] Meng J., Niu C., Xu L., Li J., Liu X., Wang X., Wu Y., Xu X., Chen W., Li Q., Zhu Z., Zhao D., Mai L. (2017). J. Am. Chem. Soc..

[cit20] Park S.-H., Bak S.-M., Kim K.-H., Jegal J.-P., Lee S.-I., Lee J., Kim K.-B. (2011). J. Mater. Chem..

[cit21] Hu H., Zhao Z., Zhou Q., Gogotsi Y., Qiu J. (2012). Carbon.

[cit22] Hu M., Ishihara S., Ariga K., Imura M., Yamauchi Y. (2013). Chem.–Eur. J..

[cit23] Hu M., Belik A. A., Imura M., Yamauchi Y. (2013). J. Am. Chem. Soc..

[cit24] Bu F., Feng X., Jiang T., Shakir I., Xu Y. (2017). Chem.–Eur. J..

[cit25] Chen S., Duan J., Ran J., Jaroniec M., Qiao S. Z. (2013). Energy Environ. Sci..

[cit26] Bu F.-X., Hu M., Zhang W., Meng Q., Xu L., Jiang D.-M., Jiang J.-S. (2015). Chem. Commun..

[cit27] Zhang B., Zheng X., Voznyy O., Comin R., Bajdich M., García-Melchor M., Han L., Xu J., Liu M., Zheng L., Gar-cía de Arquer F. P., Dinh C. T., Fan F., Yuan M., Yassitepe E., Chen N., Regier T., Liu P., Li Y., De Luna P., Janmohamed A., Xin H. L., Yang H., Vojvodic A., Sargent E. H. (2016). Science.

[cit28] Pi Y., Shao Q., Wang P., Lv F., Guo S., Guo J., Huang X. (2017). Angew. Chem., Int. Ed..

[cit29] Nai J., Lu Y., Yu L., Wang X., Lou X. W. (2017). Adv. Mater..

[cit30] Dong C., Kou T., Gao H., Peng Z., Zhang Z. (2017). Adv. Energy Mater..

