# Supplementary material for: Microwave-assisted CVD-like synthesis of dispersed monolayer/few-layer N-doped graphene encapsulated metal nanocrystals for efficient electrocatalytic oxygen evolution
Source: Chem Sci. 2018 Jul 20;9(34):7009–16. doi: 10.1039/c8sc02444h (PMC6124904; doi:10.1039/c8sc02444h)
Supplement: Supplementary file 1 [file SC-009-C8SC02444H-s001.pdf]

Electronic Supplementary Material (ESI) for Chemical Science.
This journal is © The Royal Society of Chemistry 2018

Supporting Information

Microwave-Assisted CVD-Like Synthesis of Dispered Monolayer/Few-Layer N-Doped Graphene Encapsulated Metal Nanocrystals for Efficient Electrocatalytic Oxygen Evolution

Fanxing Bu,[a] Wenshu Chen,[b] Jiajun Gu,[b] Phillips O. Agboola,[c] Najeeb Fuad Al-Khalli,[d] Imran Shakir,*[e] and Yuxi Xu*[a]

S1: Experimental section

GO synthesis and purification. Briefly, graphite (3.0 g) was added to concentrated sulfuric acid (70 mL) under stirring at room temperature, then sodium nitrate (1.5 g) was added, and the mixture was cooled to 0 °C. Under vigorous agitation, potassium permanganate (9.0 g) was added slowly to keep the temperature of the suspension lower than 20 °C. Successively, the reaction system was transferred to a 35-40 °C water bath for about 0.5 h, forming a thick paste. Then, 140 mL of water was added, and the solution was stirred for another 15 min. An additional 500 mL of water was added followed by a slow addition of 20 mL of H_2O_2 (30%), turning the color of the solution from brown to yellow. The mixture was filtered and washed with 1:10 hydrochloric acid aqueous solution (250 mL) to remove metal ions followed by repeated washing with water and centrifugation to remove the acid. The resulting solid was dispersed in water by ultrasonication for 1 h to make a GO aqueous dispersion (0.5 wt %). The obtained brown dispersion was then subjected to 30 min of centrifugation at 4000 rpm to remove any aggregates. Finally, it was purified by dialysis for one week to remove the remaining salt impurities for the following experiments.

Synthesis of Ni-Fe PBA/GO composite and Ni-Fe PBA/GO sponge. 20 μL 0.5 M $Na_4Fe(CN)_6$ solution was firstly added into 1 mL 4 mg/mL GO and sonicated for 10

min. Then 0.2 ml 0.5 M $NiCl_2$ solution was added to the above mixture and stirred for 60 min. At last, the product was centrifuged and Ni-Fe PBA/GO composite was fabricated. To prepare Ni-Fe PBA/GO sponge, the above Ni-Fe PBA/GO composite was firstly dispersed into 1 ml H_2O and then was freeze-dried by liquid N_2. For the farbication of Ni-Fe PBA, all the experimental parameters were the same with those of Ni-Fe PBA/GO except that GO was not used.

Synthesis of other MOF/GO composites and MOF/GO sponges. For the preparation of MOF/GO composite and MOF/GO sponge, only the species of metal ions and ligand ions were changed with other parameters same with those of Ni-Fe PBA/MOF composites and Ni-Fe PBA/MOF sponges. Specifically, 20 μL 0.5 M $K_3Co(CN)_6$ and 0.2 mL 0.5 M $NiCl_2$ were employed for Ni-Co PBA, 20 μL 0.5 M $K_3Co(CN)_6$ and 0.2 mL 0.5 M $CoCl_2$ were used for Co-Co PBA, 20 μL 0.5 M $Na_4Fe(CN)_6$ and 0.1 mL 0.5 M $NiCl_2$ and 0.1 ml 0.5 M $CoCl_2$ were adopted for CoNi-Fe PBA.

Synthesis of RGO and M@NC/RGO composites. For the preparation of RGO and M@NC/RGO composite, the obtained GO and MOF/GO sponges were firstly filled into one glass vial loaded with carbon cloth under Ar. Then the vials were closed and then put into a domestic microwave oven. After microwave irradiation at 1000 W for 10 s, the RGO and M@NC/RGO sponges were obtained.

Thermal conversion of Ni-Fe PBA/GO composites by tranditional programmed heat treatment. The obtained MOF/GO sponges were put into ceramic crucial, and the heating temperature was increased to 600 and 700 °C at a rate of 5 °C/min, and maintained for 2 h under argon atmosphere.

Thermal conversion of Ni-Fe PBA by tranditional programmed heat treatment. The obtained Ni-Fe PBA powders were put into ceramic crucial, and the heating temperature was increased to 700 °C at a rate of 5 °C/min, and maintained for 2 h under argon atmosphere.

Characterizations. The phase compositions of the prepared samples were monitored by X-ray diffraction (XRD), Raman spectra and X-ray photoelectron spectroscopy (XPS). XRD analysis were carried out on a D/Max 2500 X-ray diffractometer with

Cu-Kα radiation (k = 1.54 Å) with a scanning speed of 5° min^{-1} from 10° to 80°. XPS was performed on an ESCALAB 250 X-ray photoelectron spectrometer using Al Ka radiation. Raman measurements were recorded on an Invia/Reflrx Lasser Micro-Raman spectroscope (Horiba Jobin Yvon, Franch) with excitation laser beam wavelength of 532 nm. The morphologies of the products were determined on a scanning electron microscope (SEM, Zeiss Ultra-55), and a transmission electron microscope (TEM, FEI Tecnai G2 20TWIN). Nitrogen adsorption/desorption isotherms were measured at 77 K with a JW-BK132F Surface Area and Pore Size Analyzer. The specific surface area was calculated using the BET method. The pore size distribution was obtained by BJH model. The atom ratio of Fe to Ni in FeNi@NC/RGO is 1:1.7, which is determined by inductive coupled plasma emission spectrometer (ICP, iCAP 7400). The loading mass of M in M@NC/RGO composites were determined by TGA (Mettler Toledo) at a heating rate of 20 °C/min under an atmospheric environment. Since the final products after heat treatment in air are metal oxides, the loading mass of M could be determined by subtracting the mass of oxygen in metal oxides.

Electrochemical measurements. The electrochemical measurements were performed in a three-electrode system on an electrochemical workstation (CHI 760E) in KOH electrolyte. The catalysts dispersed onto a 5 mm diameter glassy carbon electrode (GC, PINE, PA, USA) were used as a working electrode, Hg/HgO and graphite rod served as the reference and counter electrodes, respectively. Typically, 5 mg of catalyst and 60 μL Nafion solution (Sigma Aldrich, 5 wt %) were dispersed in 1.94 mL ethanol solution by sonicating for 1 h to form a homogeneous ink. Then 40 μL of the dispersion was dropped onto the polished GC electrode (loading of 0.51 mg cm^{-2}). Commercial IrO_2 (99.9%, Aladdin) were used as a reference to evaluate the electrocatalytic performance of various samples. The ink of IrO_2 was prepared as the same as before and the loading is 10.4 mg cm^{-2}. All electrodes were dried before OER test. For OER tests, catalysts were firstly activated by potential cycling between 0.0 and 1.6 V *vs.* reversible hydrogen electrode (RHE) at 50 mV s^{-1} in KOH for 9 times. LSV was carried out at 5.0 mV s^{-1} in O_2 saturated KOH for the polarization curves. In

1 M KOH, E (RHE) = E (Hg/HgO) + 0.925 V. In 0.1 M KOH, E (RHE) = E (Hg/HgO) + 0.866 V. The overpotential (η) for OER was η = E (RHE) - 1.23 V. EIS was performed from 100 kHz to 0.1 Hz at a potential of 1.53 V vs. RHE with a sinusoidal voltage of 10 mV. All polarization curves were corrected with *iR*-compensation. The stability evaluations of OER were carried out by CP measurements at 10, 50, and 100 mA cm^{-2}.

S2: Figures and Tables

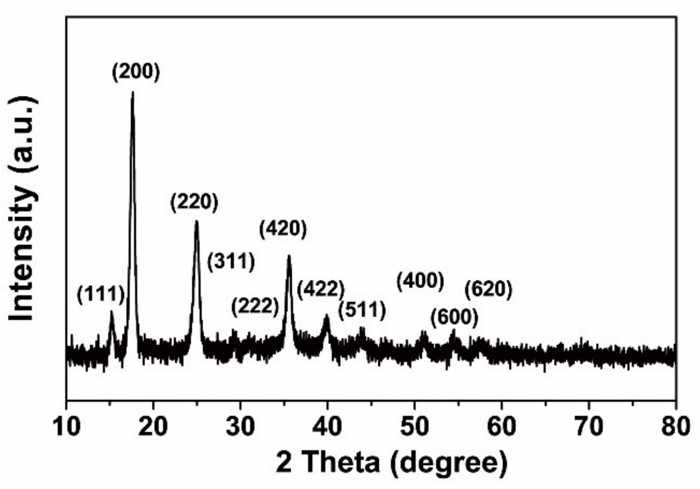

Figure S1. XRD picture of Ni-Fe PBA/GO sponge.

Table S1. Content percentage of zero-valence metal (M^0) and quaternary N among metal and N elements in different samples. This is one semi-quantitative analysis based on peak area ratio.

Sample	Fe0	Ni0	N
FeNi/RGO	30.8	35.2	19
FeNi@AC/RGO	23.6	30	23.7
FeNi@NC/RGO	37	38.2	41.2

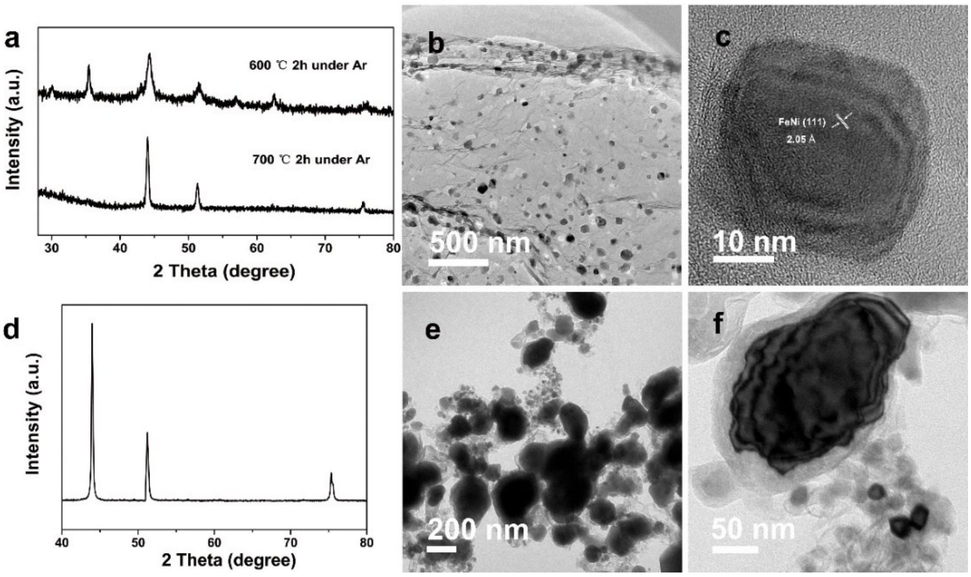

Figure S2. (a) XRD pictures and (b, c) TEM images of Ni-Fe PBA/GO derived products obtained by traditionally programmed controlled heat treatment at 700 ℃ under Ar for 2 h; (d) XRD picture and (e, f) TEM images of Ni-Fe PBA/GO derived products obtained by traditionally programmed controlled heat treatment at 700 ℃ under Ar for 2 h.

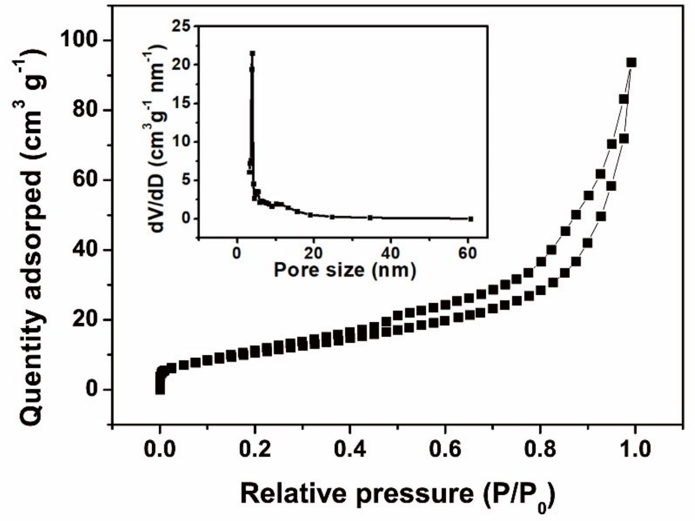

Figure S3. N_2 adsorption/desorption isotherm of FeNi@NC obtained by programmed heat treatment of Ni-Fe PBA powder. (inset: the corresponding BJH pore-size distribution curve).

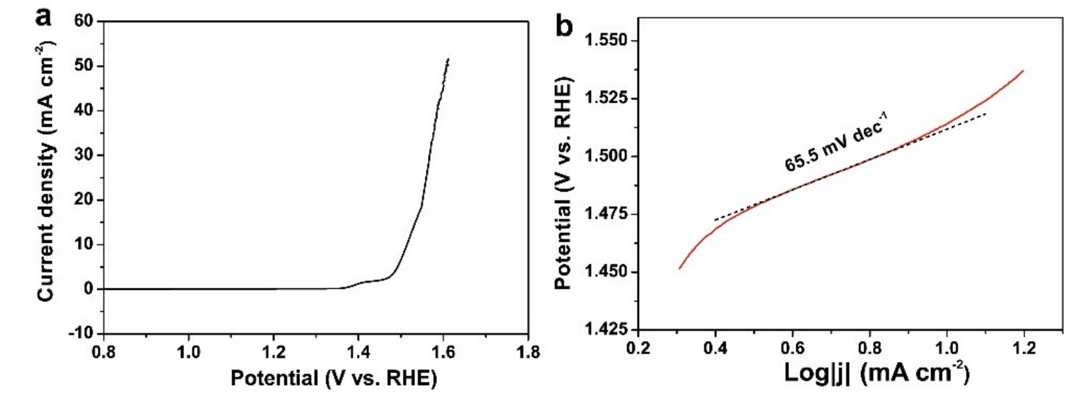

Figure S4. (a) *iR*-corrected polarization curve and (b) Tafel plot of the FeNi@NC/RGO composite measured in 0.1 M KOH.

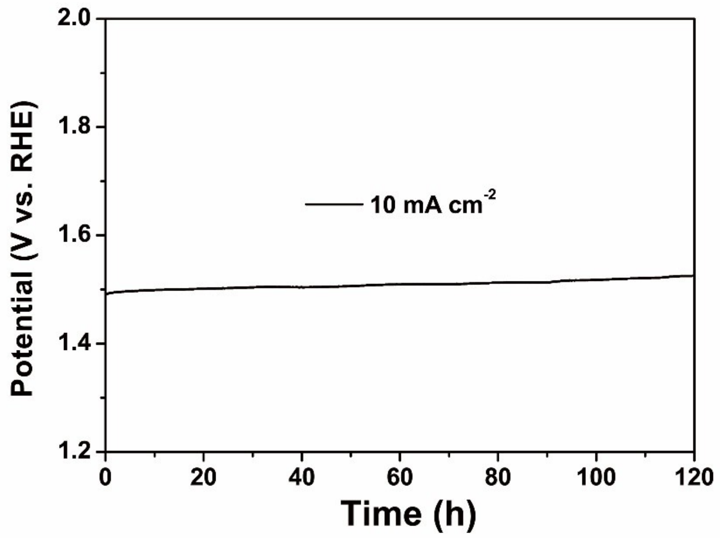

Figure S5. OER CP curve of FeNi@NC/RGO at 10 mA cm^{-2} for 120 h.

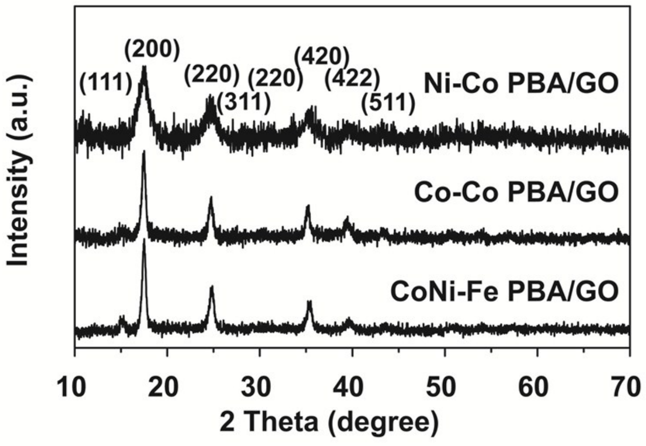

Figure S6. XRD pictures of MOF/GO composites.

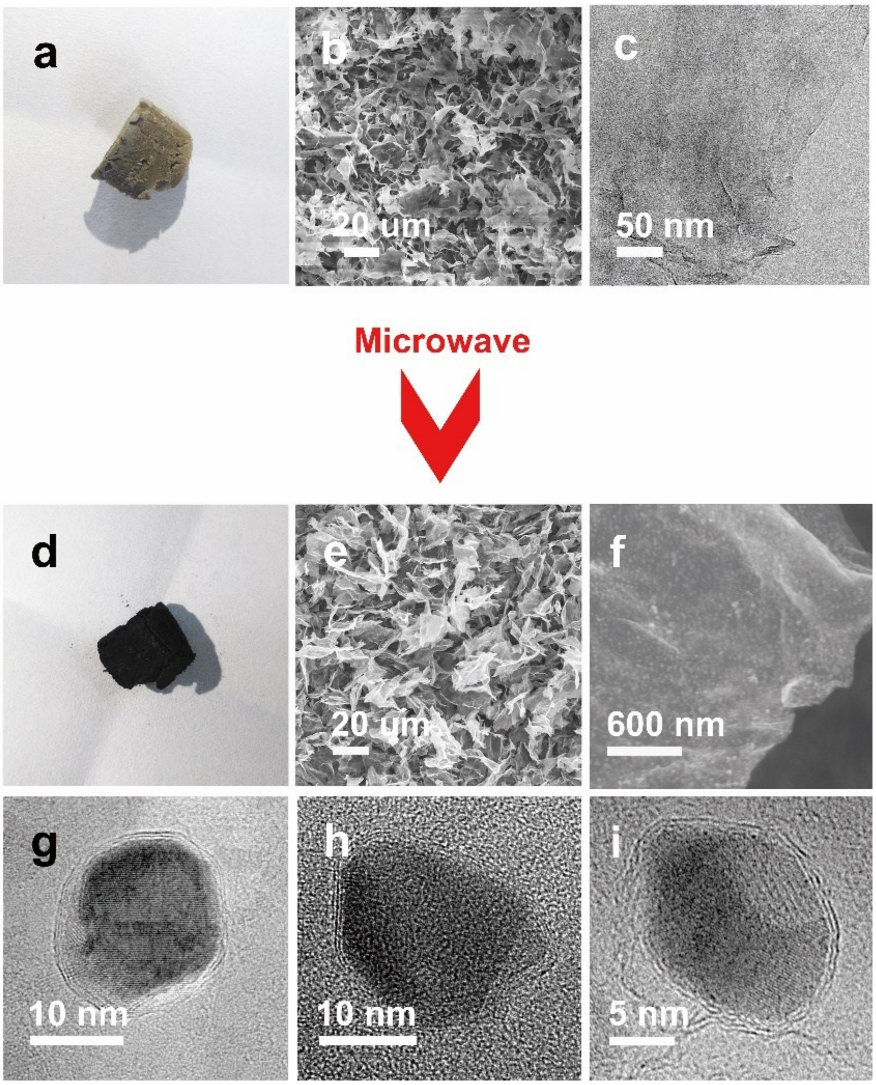

Figure S7. (a) Photograph, (b) SEM and (c) TEM pictures of Ni-Co PBA/GO. (d) Photograph, (e,f) SEM and (g-i) HRTEM pictures of CoNi@NC/RGO.

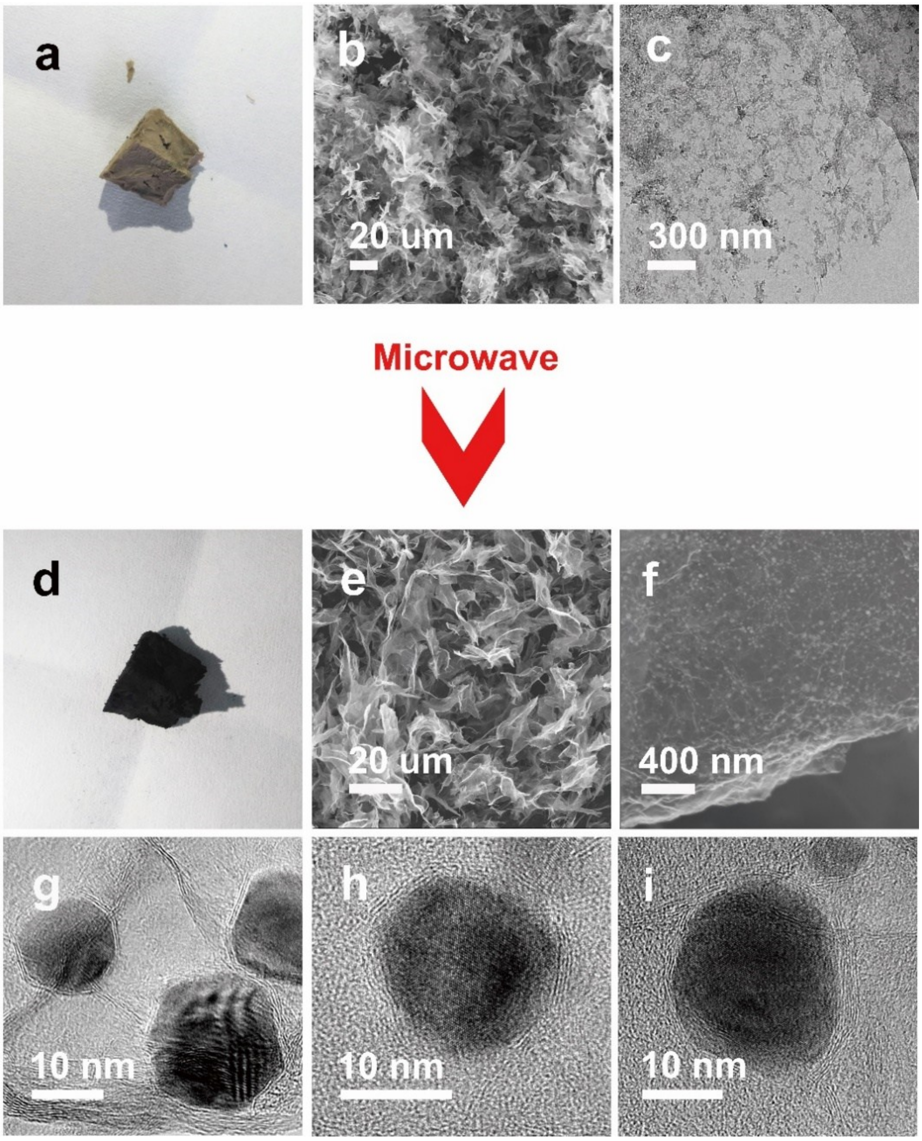

Figure S8. (a) Photograph, (b) SEM and (c) TEM pictures of Co-Co PBA/GO. (d) Photograph, (e, f) SEM and (g-i) HRTEM pictures of Co@NC/RGO.

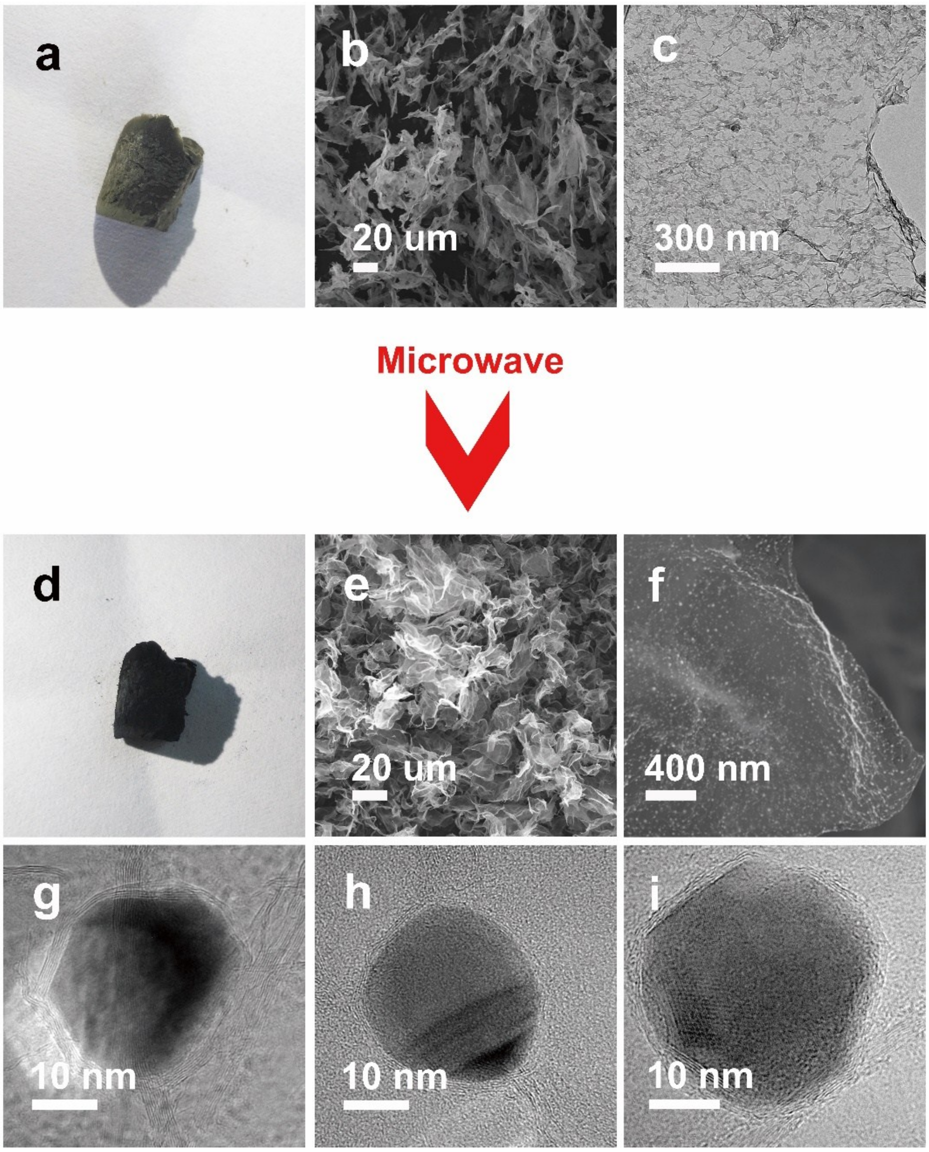

Figure S9. (a) Photograph, (b) SEM and (c) TEM pictures of CoNi-Fe PBA/GO. (d) Photograph, (e, f) SEM and (g-i) HRTEM pictures of FeCoNi@NC/RGO.

Table S2. Comparison of OER performance for FeNi@NC/RGO with other non-precious metal electrocatalysts.

Catalysts	η (mV)[a]	Tafel slope (mV dec^{-1})	Electrolyte	Counter electrode	Durability at 10 mA cm^{-2}	Reference
**FeNi@NC/RGO**	**261**	**40.7**	**1 M KOH**	**Graphite rod**	**120 h, 30 h[b]**	**This work**
	**292**	**65.5**	**0.1 M KOH**		**NA**	
p-SnNiFe	350	35	0.1M KOH	Pt sheet	5.6 h	*Nat. Commun.* **2017**, *8*, 934.
Co/NCNT	337	NA	1 M KOH	Pt wire	NA	*Angew. Chem. Int. Ed.* **2017**, *56*, 13781-13785.
Fe@N-C700	480	NA	0.1M KOH	Graphite	NA	*Nano Energy* **2015**, *13*, 387-396.
Co/N-CNTs	390	67	0.1M KOH	Pt wire	NA	*J. Mater. Chem. A* **2016**, *4*, 1694-1701.
Ni－NG	320	188.6	1 M KOH	Pt wire	12 h	*Energy Environ. Sci.* **2013**, *6*, 3693-3699.
FeNi@NC	280	70	1 M NaOH	Pt wire	16.7 h[c]	*Energy Environ. Sci.* **2016**, *9*, 123-129.
FeCoNi@NC	325	60	1 M NaOH	Pt wire	8.3 h[d]	*ACS Cataly.* **2017**, *7*, 469-479.
	428	NA	0.1 M KOH		NA	
Ni_2Fe_1-O	244	39	1 M KOH	Pt foil	6 h	*Adv. Energy Mater.* **2017**, 1701347.
	359	39	0.1 M KOH		NA	
$Fe_{0.5}Co_{0.5}Ox$	257	30.1	1 M KOH	Pt gauze	12h[e]	*Adv. Mater.* **2017**, *29,* 1701410.
$W_{0.5}Co_{0.4}Fe_{0.1}$/CC	250	32	1 M KOH	Graphite rod	>500 h	*Angew. Chem. Int. Ed.* **2017**, *56*, 4502-4506.
NiFe LDH	247	31	1 M KOH	Graphite rod	1h	*J. Am. Chem. Soc.* **2013**, *135*, 8452-8455.
	281	35	0.1 M KOH			
Co_4N/CC	257	44	1 M KOH	Pt gauze	12 h	*Angew. Chem. Int. Ed.* **2015**, *54*, 14710-14714.
Co_4N/CNW/CC	310	81	1 M KOH	Graphite plate	20 h	*J. Am. Chem. Soc.* **2016**, *138*, 10226-10231.
Fe-Ni_3C-2%	275	62	1 M KOH	Pt sheet	NA	*Angew. Chem. Int. Ed.* **2017**, *56,* 12566-12570.
Co_4Ni_1P NTs	245	61	1 M KOH	Pt wire	20 h[f]	*Adv. Funct. Mater.* **2017**, *27*, 1703455
Ni_2P	290	47	1 M KOH	Pt wire	10 h	*Energy Environ. Sci.* **2015**, *8*, 2347-2351.
$Co(S_xSe_{1-x})_2$	283	65.5	1 M KOH	Graphite rod	20 h	*Adv. Funct. Mater.* **2017**, *27*, 1701008

a. The overpotential to achieve 10 mA cm^{-2}.
b. The durability test was conducted at 10, 50 and 100 mA cm^{-2}.
c. The durability test was conducted at 40 and 100 mA cm^{-2}.
d. The durability test was conducted at 10 and 40 mA cm^{-2}.
e. The durability test was conducted at 10 and 20 mA cm^{-2}.
